# Quality and rural-urban comparison of tuberculosis care in Rivers State, Nigeria

**DOI:** 10.11604/pamj.2016.24.60.8497

**Published:** 2016-05-13

**Authors:** Charles Ibiene Tobin-West, Anastasia Isodje

**Affiliations:** 1 College of Health Sciences, University of Port Harcourt, Nigeria; 2 Department of Community Medicine, University of Port Harcourt Teaching Hospital, Port Harcourt, Nigeria

**Keywords:** Health quality, tuberculosis care, Nigeria

## Abstract

**Introduction:**

Nigeria ranks among countries with the highest burden of tuberculosis. Yet evidence continues to indicate poor treatment outcomes which have been attributed to poor quality of care. This study aims to identify some of the systemic problems in order to inform policy decisions for improved quality of services and treatment outcomes in Nigeria.

**Methods:**

A comparative assessment of the quality of TB care in rural and urban health facilities was carried out between May and June 2013, employing the Donabedian model of quality assessment. Data was analysed using the SPSS software package version 20.0. The level of significance was set at p < 0.05.

**Results:**

Health facility infrastructures were more constrained in the urban than rural settings. Both the urban and rural facilities lacked adequate facilities for infection control such as, running water, air filter respirators, hand gloves and extractor fans. Health education and HIV counselling and testing (HCT) were limited in rural facilities compared to urban facilities. Although anti-TB drugs were generally available in both settings, the DOTS strategy in patient care was completely ignored. Finally, laboratory support for diagnosis and patient monitoring was limited in the rural facilities.

**Conclusion:**

The study highlights suboptimal quality of TB care in Rivers State with limitations in health education and HCT of patients for HIV as well as laboratory support for TB care in rural health facilities. We, therefore, recommend that adequate infection control measures, strict observance of the DOTS strategy and sufficient laboratory support be provided to TB clinics in the State.

## Introduction

Tuberculosis (TB) is one of the most prevalent human infection and is the second leading cause of deaths from infectious diseases worldwide [1]. In 2012, approximately 1.4 million deaths were attributed to TB infection [2]. Nigeria ranks 10^th^ of the 22 high burden TB countries and one of the 27 high burden multidrug resistant TB (MDR-TB) countries [1]. In the year 2012, Nigeria recorded over 190,000 new cases and over 280,000 prevalent TB cases [2]. Tuberculosis is the commonest infection in people living with HIV/AIDS and is responsible for most of the mortality in this group [3]. In 2010, the number of new cases of TB in HIV infected persons was 5,100 [4]. This translated to approximately 24.3% of all new cases of TB for that year.

TB was declared a global public health emergency by the WHO in 1993 [5] and in 1995, the Directly Observed Treatment Short Course (DOTS) regimen was adopted as the key strategy in resolving the global problem [6]. Thus far, the DOTS strategy has yielded successful results in most parts of the world, leading to the interruption of TB transmission in several countries, and reduced morbidity, mortality and disability, as well as preventing the spread of drug resistant strains. It has also caused the reduction of the TB burden among People Living with HIV (PLWH) [7–9].

However, most countries in Sub-Saharan Africa have failed to meet the WHO targets which are: to achieve 70% case detection rate and 85% treatment success rate (defined as cured or treatment completed) of all detected cases. In Nigeria, the case detection and treatment success rates were among the lowest of the TB high-burden countries: 40% and 73%, respectively compared to China which had 86% and 95% [1]. The situation is even worse in Rivers State. A review of the Rivers State TB Control Program records (2001-2005) by Nwidu et al revealed a declining cure rate from 17.05% to 15.53%, which they attributed to the ineffective management of the DOTS strategy, as well as some factors that minimize access to health services in the state [10]. Failures in DOTS programs have generally been attributed to poor quality of DOTS services [3, 11].

The successful implementation of the DOTS strategy is precedent on some conditions that include: a strong political commitment, case detection through quality assured bacteriology, short-course chemotherapy, patient's adherence to treatment, adequate drug supply as well as sound reporting and recording systems [12]. When services are provided at the required standards, the health of TB patients are restored and the spread of the disease to close contacts prevented. On the other hand, substandard care results in poor treatment outcomes, persistent infectiousness as well as possible emergence and spread of drug resistant strains. Therefore, the quality of the services provided determines the likely success of the TB control programme [13]. However, evidence from studies have shown immense variation in the quality of TB care provided and this continues to plague the global TB control efforts, especially in poor resource settings like Rivers State, Nigeria. TB control programmes need therefore to ensure that services are of the highest possible quality, with a clear link between care and control, as well as within the limits of local sustainability [14].

It is against this backdrop that the Global Plan to Stop TB (2011-2015) expressed the need for operational research that will help identify and address existing systemic problems of the TB control programme, especially as it concerns the quality of services both in rural and urban settings [4]. In light of this, our study is aimed at assessing and comparing the quality of TB control services in rural and urban settings, in order to identify some of the systemic problems constraining the beneficial outcome of the DOTS strategy in TB management. Information obtained may be useful in strengthening the DOTS strategy for the improvement of tuberculosis management in the Rivers State.

## Methods

### Study area

The study was conducted in Rivers state in Southern Nigeria. The state is one of the six most populous in the country. It has a population of about 6.2 million people and over 20 ethno-linguistic groups, distributed within 23 Local Government Areas (LGA); four urban and nineteen rural. The predominant occupations in the rural area are subsistence farming and fishing. The Rivers State Tuberculosis Control Program is a unit of the Department of Public Health of the State Ministry of Health. The unit receives technical support from the German Leprosy Relief Association (GLRA) and the National TB Control Programme. There are 112 functional DOTS clinics and 22 functional microscopy centres, instead of the 216 and 54 required for optimal service delivery in the state [15].

### Theoretical framework for the study

Quality was defined by Crosby in 1979 to mean “conformance to specification” [16]. Quality assessment is a component of quality assurance that looks for sources of problems in a system and not merely bad performers. One of the earliest and prominent models used to evaluate the quality of health care services was developed by Avedis Donabedian [17]. He described health service delivery as a continuum of services. It begins with the service structure, the process of care and the end result or the outcome of care [17]. The Donabedian framework of quality assessment is aimed at determining whether inputs from the structure and process of care are appropriate to bring about a commensurate outcome. The structural attributes include: organizational structure such as supervision, training, retraining and health worker-patient ratio; and infrastructure such as waiting areas, examination rooms, equipment and drugs [18]. The process of care in the Donabedian context tends to determine whether the right procedures were being followed and that the patient-provider interactions were appropriate. The outcome measures could be any of the following: treatment outcome, patient satisfaction or the impact of the service in the community served [18].

### Study design

The study was carried out using a comparative cross-sectional design, which assessed and compared the quality of DOTS services provided in rural and urban TB DOTS facilities. It employed qualitative and quantitative research methods in collecting data from patients as well as the health facilities.

### Study population

Study population included all patients ages 15 and above, with pulmonary TB who received treatment from the selected TB/DOTS clinics in the sampled LGAs, while the facilities assessed were those that provided DOTS services for patients with TB according to the National guidelines.

### Eligibility criteria

Health care facilities that provided diagnosis and treatment for TB patients, according to the National guideline and TB patients managed as out-patients for more than four weeks were included. On the other hand, TB patients who were transferred-in less than four weeks prior to commencement of the study, as well as patients admitted in a health facility for co-morbidities or complications of TB were excluded.

### Sample size determination

The minimum sample for the study was determined using a formula for calculating sample sizes for comparison of two proportions [19]. The computation was based on a study carried out in Egypt, which revealed that 78% of the participants who completed treatment, were satisfied with the overall services received [20]. A minimum sample of 117 patients from the rural and urban facilities each was considered adequate after adjustment for attrition of 5%.

**Sampling technique:** A multi-stage sampling technique was used to select the health care facilities for the study.

**Stage I: selection of LGAs:** Two rural and two urban LGAs were selected by a simple random method from the existing four urban and nineteen rural LGAs in the State. The urban LGAs were: Obio-Akpor and Port Harcourt city LGAs, while the rural were Khana and Gokana LGAs.

**Stage II: facility selection:** A minimum of 25% of the health facilities was included in the study based on WHO recommendation for such evaluative studies [21]; three were selected from Obio-Akpor LGA, two from Port Harcourt city LGA, while two were selected from Khana LGA and one the Gokana LGA.

**Stage III: selection of participants:** Sampling proportionate to size was used to select patients for the study. All eligible and consenting patients were serially enlisted on clinic days until the desired sample sizes were attained.

### Data collection

**Figure 1:**
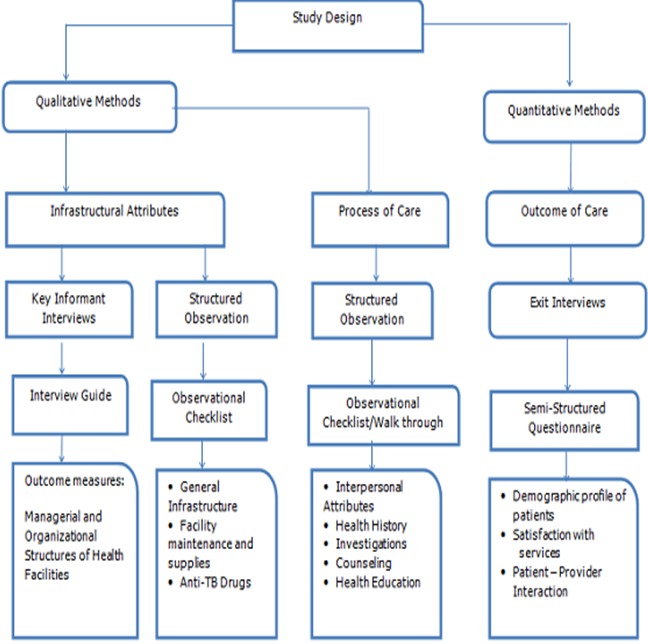
Schematic diagram for data collection

The data tools were pre-tested in a DOTS facility not selected for the study to ensure face validity of the data. The tools used were: a Semi-structured interview guide for TB Focal persons, an Observational checklist for Infrastructural facilities and Process of care and a Semi-structured questionnaire for the Outcome of care. A summary diagram for data collection is presented in Figure 1. Data was collected over a six week period, between May and June 2013.

### Key informant interviews of TB Focal persons

A semi-structured interview guide was used to generate information from key informants (TB Focal persons). The interview guide contained 13 questions which sought information on the managerial and organizational attributes of the TB DOTS services. It covered such topics as staffing, training and re-training, supervision and availability of logistics for service delivery and more. A total of eight facility-based TB Focal persons were interviewed; five females and three males. Three of the interviewees were from the rural facilities while five were from urban facilities.

### Structured observation of infrastructural facilities

A checklist adapted from the National Tuberculosis and Leprosy Control Programme (NTBLCP) was used to generate information on facility structures. The tool had four major sections: general infrastructure which comprised of waiting area, examination room, availability of toilet facilities and availability of a functional laboratory. Others were Facility maintenance; Supplies; and Anti-TB drugs. It was administered via a walk-through survey of the facilities. Scores were graded on a three point scale as satisfactory, fair and unsatisfactory.

### Structured observation of the process of TB care

Direct observation of work processes, was done using a tool adapted from the NTBLCP workers manual. The tool was in two sections: Interpersonal attributes and Technical aspects of patient care. The measurement of these attributes was also graded as: satisfactory, fairly satisfactory and unsatisfactory. The Technical aspect of patient care involved: general history taking, investigations and health education/counselling.

### Data collection for the outcome of TB care

A modified Ware's PSQ-III questionnaire was adapted and used to generate data on the outcome of TB care [22]. The questionnaire was in two parts: the first part focused on the patients socio-demographics; and the second part assessed their satisfaction with the services provided as proxy measure for the outcome of care. The second section was on patient satisfaction. The questions focused on patients’ satisfaction in three areas: organizational issues; facility equipment and supplies; and patient-provider interaction. Responses were graded on a three-point Likert's scale: satisfied, fair and dissatisfied. The questionnaires were administered through Exit Interviews of patients who had just finished using services at the health facilities.

### Data analysis

**Key informant interviews:** The key informant interviews were transcribed and coded. The transcript contained every question asked; verbalizations such as pauses, hesitation and other audible behaviour were also transcribed. Patterns and themes were identified and these were analyzed using the thematic content analytical approach [23].

**Infrastructural attributes:** The variables for assessing the infrastructural attributes were ranked as: satisfactory, fairly satisfactory and unsatisfactory. To determine a satisfactory score in each of the variables, the composite score was determined and the attainment of 50% was classified as satisfactory and less than 50% was classified as unsatisfactory.

**Process of care:** Observations of the process of care were rated as satisfactory, fairly satisfactory or unsatisfactory. The variables observed were: patient-provider interaction, history of cough and health education/counselling. Attainment of 50% of the composite scores was classified as satisfactory and less than 50% was classified as unsatisfactory.

**Outcome of care:** Data was analyzed with SPSS version 20.0 software package. The Chi-square test was performed for categorical variables to determine any differences in the study settings. The level of significance was set at *p < 0.05*. Dichotomous variables were created from the original three-point Likert's Scale such that the two points at the favourable end of the satisfaction scale were recoded as “satisfied”, while the three Likert's points at the unfavourable end of the satisfaction scale were recorded as “dissatisfied”. Logistic regression was also computed to determine the association between explanatory variables and overall satisfaction.

### Ethical approval

The Ethics Committee of the University of Port Harcourt Teaching Hospital approved the study. Written permission to conduct the study was received from the Rivers State Primary Health Care Management Board, while written informed consent was obtained from all participants. Participants were all assured of strict confidentiality of their responses.

## Results

### Physical and laboratory infrastructure

Three out of the five urban facilities, 3 (60.0%) had adequate space, ventilation and sunlight in the clinic waiting areas compared with two out of three in the rural health facilities 2 (66.7%). In the examination rooms, three urban facilities met the minimum criteria for privacy and toilet facilities as against one facility in the rural area. More of the rural facilities, 2 (66.7%) had minimal air recirculation compared to the urban, where all facilities failed to meet the minimum criteria. Only one facility, 1 (20.0%) in urban setting had running water for hand washing in the consulting rooms. All the facilities, both urban and rural did not have the facilities for infection control, such as air filters, respirators, hand gloves and extractor fans.

In terms of facility maintenance, it was observed that 4 (80.0%) of facilities in the urban areas met the minimum criteria for walls and floor maintenance, while all met the criteria for cleanliness of the floor. Nevertheless, only 2 (40.0%) of the facilities met the criteria for cleanliness of the toilets, while all the facilities in the rural areas had dirty toilets. Furthermore, while 3(60.0%) of the laboratories in the urban health facilities were functional, only 1(33.3%) was among the rural health facilities (Table 1).

**Table 1:** Facilities with the recommended infrastructure and laboratory services

General infrastructure	Location of facilities	
	Urban n = 5 (%)	Rural n = 3 (%)
***Waiting area***		
Space	3 (60.0)	1 (33.3)
Ventilation	3 (60.0)	2 (66.7)
Sunlight	3 (60.0)	2 (66.7)
***Examination room***		
Privacy	3 (60.0)	1 (33.3)
Water for washing hands	1 (20.0)	0 (0.0)
Hand gloves	0 (0.0)	0 (0.0)
Air filter respirators	0 (0.0)	0 (0.0)
Extractor fans	0 (0.0)	0 (0.0)
Avoidance of air re-circulation	0 (0.0)	2 (66.7)
***State of Maintenance of facilities***		
***Floor and walls***		
No cracks	4 (80.0)	1 (33.3)
No holes	4 (80.0)	2 (66.7)
***Cleanliness***		
No litter on the floor	5 (100.0)	2 (66.7)
No stains on the floor	2 (40.0)	1 (33.3)
***Toilet facility***		
No faeces littering toilet	2 (40.0)	0 (0.0)
No foul odour	2 (40.0)	0 (0.0)
***Functional laboratory***		
Functional laboratory	3 (60.0)	1 (33.3)
Air filter masks	0 (0.0)	0 (0.0)
Hand gloves	2 (40.0)	1 (33.3)
Water for hand washing	3 (60.0)	1 (33.3)

### Process of care

With regard to working materials, all facilities in the rural areas had at least six out of the eight (75.0%) essential materials for TB care. These included the TB workers manual, referral and transfer forms, sputum examination request forms, case finding, treatment outcome and other TB control activity report forms, etc. However, only four out of the eight, (50.0%) of materials required were available in the urban facilities. All the facilities had anti-TB drugs (Table 2). Furthermore, in 24 (80.0%) of the observations in urban facilities and 30 (100%) in rural facilities, health care workers were polite and explained the diagnosis to their patients. Nevertheless, none of the patients in both settings had satisfactory privacy (Table 3).

**Table 2:** Availability of materials and drugs

Materials	Location of facilities	
	Urban n = 5 (%)	Rural n = 3 (%)
NTLCP workers manual	5 (100.0)	3 (100.0)
TB unit registry	5 (100.0)	3 (100.0)
TB referral & transfer form	4 (80.0)	3 (100.0)
TB sputum examination request form	5 (100.0)	3 (100.0)
Quarterly case finding, treatment outcome and other TB control activity report form	4 (80.0)	2 (66.7)
TB flip chart in consulting room	4 (80.0)	3 (100.0)
TB posters in waiting area	4 (80.0)	2 (66.7)
Functional weighing scale	5 (100.0)	3 (100.0)
***Anti- TB drugs***		
Rifampicin/Isonizid/Pyrazinamide (RHZ)	5 (100.0)	3 (100.0)
Ethambutol/Isoniazide (EH)	5 (100.0)	3 (100.0)
Streptomycin (STM)	5 (100.0)	3 (100.0)
Complete drugs for each patient	5 (100.0)	3 (100.0)
Storage area for drugs	3 (60.0)	1 (33.3)

**Table 3:** Patient-provider interaction in the process of TB care

Variable	Location of facilities	
	Urban n = 30	Rural n = 30
	Satisfactory	Satisfactory
	n (%)	n (%)
Politeness/ greeted client	24 (80.0)	30 (100.0)
Made patient comfortable	5 (16.7)	12 (40.0)
Listened to patient	0 (0.0)	2 (6.7)
Asked about patient's concerns	1 (3.3)	5 (16.7)
Privacy	0 (0.0)	0 (0.0)
Explained procedures	15 (50.0)	9 (30.0)
Explained diagnosis	22 (73.3)	28 (93.3)
Explained side effects of drugs	15 (50.0)	13 (43.3)

Health services like history taking, was satisfactory in 29 (96.7%) of observations in urban and 28(93.3%) of rural centres. However, history about occupation and overcrowding were poorly taken in both settings (<35%). HIV counselling and testing (HCT) was equally low: 16.7% in urban and 6.7% in rural settings. The Directly Observed Therapy (DOTS) strategy was not implemented in any of the facilities assessed. Health education and counselling were satisfactory in only 3(10.0%) of observations. Patient-provider interaction was satisfactory in only 12 (40.0%) in the urban facilities compared to 18 (60.0%) in the rural facilities. For the composite score for adequacy of TB care, 15 (50.0%) of the observations in rural facilities met the criteria compared to only 4 (13.3%) in urban facilities (*p = 0. 005*) (Table 4).

**Table 4:** Number (%) of TB care processes with minimum requirement

Variables	Number of satisfactory observation		χ^2^/ Fishers exact	p-value
	Urban n= 30 n(%)	Rural n= 30 n(%)		
Patient-provider interactions^1^	12 (40.0)	18 (60.0)	2.400	0.121
History taking^2^	29 (96.7)	28 (93.3)	0.351	1.000
Health education/Counseling^3^	3 (10.0)	1 (3.0)	1.071	0.612
Adequate TB care process^4^	4 (13.3)	15 (50.0)	9.320	0.005
HCT	5(16.7)	2(6.7)		

1 The maximum attainable score was 16; therefore overall satisfaction was coded as ≥ 12 while dissatisfaction was coded as <12

2 The maximum attainable score was 7; therefore overall satisfaction was coded as ≥ 5 while dissatisfaction was coded as < 5

3 The maximum attainable score was 10; therefore overall satisfaction was coded as ≥ 7 while dissatisfaction was coded as <7

4 The maximum attainable score was 33; therefore overall satisfaction was a summation of satisfaction in sub-components above and coded as ≥ 24 while dissatisfaction was coded as <24

### Patient monitoring and defaulter tracing

All key Informants acknowledged the importance of patient monitoring and follow up for assessing improvement. They mentioned the role of sputum AFB follow-up tests and monthly weight measurement of patients in determining patients’ response to treatment. They all mentioned defaulter tracing as an important component of patient monitoring, but only a few said they were able to carry out home visits. However, they all had alternative means of tracing these patients. These included phone calls, use of treatment supporters and community volunteers. A few said that in the facilities where they work, money was provided occasionally for home visits to patients.

One TB Focal Person mentioned: “*An NGO is piloting the use of community volunteers for defaulter tracing so it is helping us. They know these patients because they live in the same community. So whenever we have not seen a patient for a while depending on the phase of treatment (intensive or continuation), we ask them to help us*” (Male, rural).

Another respondent reported: “*We tell each patient to come with a treatment supporter. The treatment supporters are taught how to tick the patient's cards and bring or encourage patient's to come for monthly weighing and sputum tests. So they are the ones I call when I did not see the patients for a while*” (Female, urban).

### Drug supplies and shortage

On drug supplies and shortages, all the Focal persons reported that drugs were supplied on a regular basis. Most said they had not experienced drug stock-out over a long period of time. They said that the main strategy they adopted was to ensure that each patient had their complete treatment package of drugs at commencement of treatment. However, when there were occasional drug shortages, some health workers ask the patients to wait for a while. One affirmed: *“When we experience shortages, we ask patients to wait. We collect their names, phone numbers and home addresses, so that once we receive supplies, we contact them and each patient is provided with a pack. If we don't have packs we don't start treatment”* (Male, rural).

***Outcome of care:*** Generally speaking, most patients were satisfied with the interactions with their care providers, especially for those attending the rural health centres (*p = 0.005*) (Table 5). Nevertheless, logistic regression analysis did not confirm any association between independent variables responsible for the overall satisfaction noticed (Table 5).

**Table 5:** Patients’ satisfaction with service providers

*Patient-provider interaction*	Urban	Rural	χ^2^/ Fishers exact	p= value
	Satisfied n=125 (%)	Satisfied n=119 (%)		
The respect given to you by Health Worker	117 (93.6)	119 (100.0)	7.874	0.005
Ability to discuss problem on your health with provider	116 (92.8)	113 (95.0)	0.492	0.483
Amount of explanation about the problem or treatment	119 (95.2)	111 (93.3)	0.417	0.519
The Health Workers skill and ability to treat you	118 (94.4)	115 (96.6)	0.710	0.400
Effectiveness of the drugs	120 (96.0)	113 (95.0)	0.154	0.695
Examination and treatment provided	119 (95.2)	112 (94.1)	0.142	0.707
Privacy from others seeing you being examined	120 (96.0)	109 (91.6)	2.05	0.152
Privacy from others hearing your discussion	117 (93.6)	108 (90.8)	0.687	0.407

## Discussion

### Quality of structural facilities

Most of the health facilities in both the rural and urban areas had sufficient waiting areas for patients. This was quite commendable as they provided a comfortable environment for patients. They compared favourably with what obtains in some other settings in Africa [5, 9]. The size of the waiting area has a role to play in the spread of TB infection as well as the prevention of re-infection of patients [24]. However, patient privacy was poor in most of the rural health facilities which was unacceptable. Lack of privacy might compromise the uptake of such services as point–of-care testing for HIV and patients’ receptiveness to the health care provider. This result was similar to what was reported in the studies in South Africa and Ethiopia [9, 25]. Patients’ privacy is an important aspect of TB care, especially as TB is associated with stigma. It therefore deserves serious attention.

Meanwhile, all the health facilities, both urban and rural lacked amenities for infection control such as, running water, air filter respirators, hand gloves and extractor fans. Only one of the eight facilities assessed had water for washing of hands in the examination room. These deficiencies have grave implications for the spread of TB and are contrary to what obtains in TB clinics in Thailand [26] and Canada [27]. Infection control measures help to minimise nosocomial infections in patients and reduces the risk of occupational hazard posed by repeated exposure of health workers to tuberculosis bacilli [24]. Substandard infection control measures can result in poor treatment prognosis, disease progression with potential transmission risks to family and health workers. Furthermore, may result in the development of drug resistant strains [28].

Most of the urban facilities had functional laboratories compared to the rural facilities. Paucity of laboratories may limit the ability of TB Control Programmes in realizing the case detection rate target of 70% for sputum smear-positive cases stipulated by WHO [2] and may also compromise the process for monitoring patient's progress in clinical settings [14]. The finding was similar to what obtained in Sidama Ethiopia [5] but different from the report of Girma et al in six health facilities of Afar region, also in Ethiopia, where a higher proportion of the rural facilities had functional laboratories [9].

All the facilities had full complements of anti-TB drugs and this was commendable, because the anti-TB drugs are key to the success of TB care [7]. Availability of drugs is made possible with donor support for tuberculosis control in Nigeria by GLRA as well as the recent commencement of the shorter six month treatment regimen, making more drugs available [14].

### Quality of process of care

Patient-provider interactions were more satisfactory in the rural health care facilities than in the urban facilities. The higher workload in urban health facilities as a result of the higher prevalence of TB in the urban areas of the state may have accounted for this finding [10]. Nevertheless, patient-provider interaction is a key predictor of patient satisfaction and subsequent adherence to the treatment plan. The finding was, however, not different from what has been reported in similar studies in Ethiopia and Tanzania [9, 29]. It is only natural that good rapport between the health care workers and the patients are more likely to lead to patient compliance with treatment regimens with resultant improvement in treatment outcomes.

Overall, health education and HCT were low in rural settings, while the DOTS strategy was completely ignored in both the urban and rural clinics. The importance of health education for TB patients with regard to the spread of the infection and the value of adherence counseling and completion of treatment cannot be overemphasized. Poor health education of TB patients was found to be a predictor of treatment failure, and in fact contributed to an 11-fold failure risk in a study carried out in Egypt [30]. Similarly, the non-implementation of HCT negates the provision of the National policy on HIV/AIDS, knowing that tuberculosis is the commonest opportunistic infection affecting people living with HIV/AIDS and that at least 50% of TB patient are infected with HIV in Nigeria [3]. Similar poor patient education was also found by Rashmir and Vijaykumar, in India [31], where they reported that only 22% of service providers educated their patients on the spread and prevention of TB. Furthermore, none implementation of the DOTS strategy defeats the laudable objective of WHO of achieving 85% treatment success rate of all detected TB cases, in order to control the spread of the infection [6].

### Outcome of care

In all, about 95.2% of patients from urban health facilities and 99.2% from rural health facilities were satisfied with the services provided by the health care workers. This was quite commendable because it has been established that satisfaction with service provision is a strong determinant of adherence and treatment completion which are key for TB control [11]. The result was similar to what was observed in rural and urban health care facilities in Ethiopia, India and Egypt [9, 31, 30]. However, some degree of dissatisfaction was reported in some urban settings in terms of the long waiting time in the clinics, uncomfortable waiting area, and the occasional stock out of medicines etc [9, 30].

### Study limitation

The study was facility-based and may thus be prone to courtesy bias, as patients may respond favorably at Exit interviews for fear of being victimized subsequently. However, patients were reassured of confidentiality as their names were not recorded on the questionnaires.

## Conclusion

The study highlights suboptimal quality of TB care in Rivers State, especially as it regards non-adherence to the DOTS strategy and limited facilities for infection control such as, running water, air filter respirators, hand gloves and extractor fans. It further highlights weaknesses in HCT of patients for HIV, and limited laboratory support in rural health facilities compared to urban facilities. We therefore recommend the following measures: in setting up TB clinics, minimum requirements for infection control should be made available in sufficient quantities on a sustainable basis; all mechanisms necessary to ensure that treatment are directly observed should be put in place, while health care providers must ensure compliance; adequate laboratory support should be provided for diagnostics and treatment monitoring.

### What is known about this topic

- The quality of DOTS plays a significant role in the success of the STOP TB program and this has resulted in the improvement of treatment outcome in regions other than Africa;
- Nigeria has one of the lowest case detection and treatment success rates of the 22 high burden countries, despite many years of DOTS implementation;
- The Global Plan to Stop TB 2011-2015 acknowledged a gap in previous research areas on TB and expressed an urgent need for operational research that will help identify and address existing systemic problems in the TB control programmes.

### What this study adds

- Information on the inadequacy of infrastructure for infection control in the delivery of DOTS/TB care in Rivers State, Nigeria;
- Information on the non-adherence of healthcare providers to the WHO/DOTS strategy in TB control;
- Information on rural-urban disparity in patient satisfaction with the process of care for TB.
